# An investigation of reconstituted terlipressin infusion stability for use in hepatorenal syndrome

**DOI:** 10.1038/s41598-020-78044-4

**Published:** 2020-12-03

**Authors:** Thi Ngoc Nhieu Bui, Su Sandar, Giuseppe Luna, Jasmine Beaman, Bruce Sunderland, Petra Czarniak

**Affiliations:** 1 School of Pharmacy and Biomedical Sciences, Faculty of Health Sciences, Curtin University, Kent St, Bentley, WA 6102 Australia; 2 Pharmacy Department, Sir Charles Gairdner Hospital, Nedlands, WA 6009 Australia

**Keywords:** Hepatology, Translational research, Preclinical research, Physical chemistry

## Abstract

Hepatorenal syndrome (HRS) is a fatal complication of renal dysfunction associated with ascites, liver failure and advanced cirrhosis. Although the best option for long-term survival is liver transplantation, in the critical acute phase, vasoconstrictors are considered first-line supportive agents. Terlipressin is the most widely used vasoconstrictor globally but owing to its short elimination half-life, it is usually administered six hourly by slow intravenous bolus injection. This requires patients to remain in hospital, increasing hospital bed costs and affecting their quality of life. An alternative option for administration of terlipressin is as a continuous infusion using an elastomeric infusor device in the patient’s home. However, stability data on terlipressin in elastomeric infusor devices is lacking. This research aimed to evaluate the stability of terlipressin reconstituted in infusor devices for up to 7 days at 2–8 °C and subsequently at 22.5 °C for 24 h, to mimic home storage and administration temperatures. We report that terlipressin was physically and chemically stable under these conditions; all reconstituted infusor concentrations retained above 90% of the original concentration over the test conditions. No colour change or precipitation in the solutions were evident.

## Introduction

Hepatorenal syndrome (HRS) is a fatal complication of renal dysfunction associated with ascites, liver failure and advanced cirrhosis^1^. It is characterised by functional renal impairment due to vasoconstriction of the renal arteries and severe vasodilation of the splanchnic arteries, leading to decreased arterial blood volume and arterial pressure^1^. There are two forms of HRS: type 1 is characterised by an acute progressive decrease in kidney function with a median survival time of 2 weeks without treatment, whereas type 2, being associated with less severe kidney failure, is more stable and without treatment, has a median survival of about 6 months^1,2^. The best option for long term survival is liver transplantation^3^. In the critical acute phase, vasoconstrictors such as noradrenaline, dopamine and terlipressin, are considered to be first-line supportive treatment^4^. Terlipressin is the most widely used vasoconstrictor globally^5^. Studies have reported that terlipressin is more effective in improving renal function in patients with HRS when compared to other drugs and placebo^6–10^.

Terlipressin, a synthetic prodrug of vasopressin^11^, is converted to the biologically active lysine-vasopressin by enzymatic cleavage^12^. Lysine-vasopressin activates vasopressin V1 receptors found in smooth muscle on the splanchnic arterial vasculature which causes vasoconstriction^12^. Splanchnic and extra-renal vasoconstriction increases renal blood flow and has beneficial effects on HRS^13^.

Terlipressin is available commercially as a solution for injection (Glypressin)^11^ and powder reconstituted for injection (Lucassin)^14^. Both forms have a shelf life of 2 years when stored in the original container at 2–8 °C^11,14^. Reconstituted Lucassin lyophilized powder for injection is reported as stable in a refrigerator at 2–8 °C for up to 24 h^14^. For patients awaiting a liver transplant this short shelf-life currently requires them to remain in hospital. Continuous administration using a 24-h infusor device would permit home administration allowing the patient to be ambulatory, with improved quality of life and also reduce hospital in-patient costs. A recent study by Cavallin et al.^15^ reported that terlipressin administered by continuous infusion was better tolerated and was also effective at doses lower than those required for intravenous bolus administration.

Currently, there is no data available on the stability of terlipressin in elastomeric infusor devices suitable for 24 h continuous infusion. This research aimed to evaluate the stability of terlipressin dispensed in infusor devices for up to 7 days at 2–8 °C and subsequently 22.5 °C for 24 h, to provide evidence for the potential use of terlipressin as a ‘hospital in the home (HITH)’ treatment while patients await a liver transplantation.

## Materials and methods

### Materials

Analytical grade terlipressin was purchased from Med Chem Express (lot 21425, expiry 11/2020; USA). Commercial terlipressin powder (Lucassin) was purchased from Mallinckrodt Pharmaceutical (lot 870403F, expiry 03/2021, AU).

High Performance Liquid Chromatography (HPLC) used a mobile phase consisting of acetonitrile (lot 180372; Fisher Chemical, USA), potassium dihydrogen orthophosphate (batch number 1809274114, expiry 09/2021; Ajax Finechem, VIC, Australia) and orthophosphoric acid 85% (batch number 1076916; Thermo Fisher Scientific, WA, Australia). Water was accessed through a MilliQ Ultrapure Water System (Merck, VIC, Australia) consisting of a four-bowl ultrapure cartridge kit with a conductivity of 0.05 µS, and was used to prepare buffers and standards^16^.

The HPLC was a Shimadzu Prominence LC-20AT consisting of a Shimadzu Prominence SIL-20ACHT Auto Sampler, a Shimadzu Prominence SPD20A UV Wavelength Detector (Shimadzu Corp, Kyoto, Japan), a Shimadzu DGU-20AC5R Degassing Unit, an Apollo C18 reverse phase column (150 × 4.6 mm, 5 µ particle size; lot 50629864), and Lab Solutions Version 5.85 analytical software (Shimadzu Corp, Kyoto, Japan)^16^.

Forced degradation studies used hydrochloric acid 36% analytical reagent (Fisher Scientific, UK), hydrogen peroxide 30% analytical-grade (batch number 16082255558; Ajax Finechem Pty Ltd, NSW, Australia), sodium hydroxide pellets (Chem Supply Pty Ltd, SA, Australia) and pH 7 buffer prepared from sodium dihydrogen orthophosphate monohydrate (batch number 16256; Meck, VIC, Australia), and di-sodium hydrogen orthophosphate anhydrous (batch number 11747; BDH Chemicals, VIC, Australia) to test the stability of commercial terlipressin. A Werke EH4.2 water bath (IKA, Germany) was used to maintain water at a constant temperature 50 °C (± 0.2 °C); temperature was measured by a Brannan 76 mm immersion thermometer (Brannan, UK)^16^.

The elastomeric infusion device was a Baxter FolFusor SV system 2.5 mL/h (lot number 18F044; Baxter Healthcare, USA). Sodium chloride 0.9% (batch number S16F1, expiry 6/2020; Baxter Healthcare, NSW, Australia) was used as a solvent for preparation of commercial terlipressin. A pH meter (Hanna Instruments, USA) was used to adjust pH and for pH measurements of terlipressin solution in the infusors.

### Assay methodology

Terlipressin was assayed by a HPLC method adapted from the British Pharmacopoeia (BP) 2019^17^. The mobile phase consisted of a mixture of phosphate buffer pH adjusted to 3.5 and acetonitrile. The buffer was filtered through a 0.45 μm nylon membrane filter (lot 020415; Altech Chemical Ltd, WA, Australia). Linear gradient elution was used with a flow rate of 1.0 mL/min and injection volume of 20 μL. The wavelength was 210 nm and the analysis time was 32 min with mobile phase elution as detailed in Table 1.

**Table 1:** Concentrations of mobile phase (phosphate buffer and acetonitrile) using a programmed elution over 32 min.

Time (minutes)	Concentration of mobile phase	
	% A (phosphate buffer)	% B (acetonitrile)
0	95	5
2	95	5
30	75	25
32	75	25

The assay linearity was performed at terlipressin concentrations between 0.002 and 0.38 mg/mL and intra-day and inter-day method precision was performed by injecting the 0.05 mg/mL standard solution six times. The data from two consecutive days were collected for interday comparison. The precision was determined as RSD = $\frac{s}{x}$ * 100 where RSD = relative standard deviation (precision); s = standard deviation and x = mean of the peak area values.

Forced degradation studies were evaluated with commercial terlipressin 0.17 mg/mL and hydrochloric acid 0.2 M, hydrogen peroxide 6%, aqueous solution (pH 4.9) and phosphate pH 7.0 buffer. The phosphate pH 7.0 buffer was made from disodium phosphate 3.3 mM, and sodium phosphate dibasic heptahydrate 2.1 mM. Terlipressin and other solutions in 5 mL volumetric flasks were transferred into vials and equilibrated in a water bath at 50 °C. Samples were taken at 0, 35, 70, 120 and 180 min. The hydrochloric acid 0.2 M was neutralized using sodium hydroxide 0.2 M prior to analysis.

### Assay of commercial terlipressin

The solutions for the stability studies were prepared using commercial terlipressin (Lucassin) containing 0.85 mg of terlipressin reconstituted in 5 mL of normal saline (based on the manufacturer’s ‘reconstitution’ instructions. Further, the product information also stated Lucassin is incompatible with dextrose solutions)^14^. Four ampoules of Lucassin were reconstituted each with 5 mL of 0.9% sodium chloride solution. The combined solutions were diluted to 100 mL with 0.9% sodium chloride solution in a 100 mL volumetric flask and transferred to an infusion device. The solution was prepared in the same way as above for all infusion devices. The luer-lock of each infusor was removed and a clamp was used to close and control the flow of solution from the infusors.

On the first day (t = 0), approximately 12 mL was withdrawn from each of the three infusors and separated into two glass vials, each of which contained 6 mL. From each infusor, one vial was used for the HPLC assay, with the remainder of the vial contents used to measure pH. The second vial was kept in the stability chamber at 22.5 °C for 24 h prior to assay to determine the concentration after 24 h in the chamber. The temperature used for the stability chamber was 22.5 °C and was determined in a previous study by measuring average temperature when an infusor was worn by several members of the research team, in a carrying pouch for 24 h, with ice packs changed every 8 h^16^. The same procedure (without pH measurement) was adopted on Days 2 and 3 (24 and 48 h). For the fourth day (t = 72 h) infusor, 6 mL of solution was taken for stability determination only and not placed in the 22.5 °C cabinet. All of the infusors and ampoules were stored in a refrigerator at 2–8 °C (actual temperature 6.7 °C [range 5.6–6.9 °C] except when samples were transferred to the stability chamber at 22.5 °C. On the seventh day (t = 144 h), the procedure was followed in the same way as the first day. All assays were carried out in triplicate.

The pH of those solutions was measured at 24 and 168 h. The solution from infusor 4 was only used to measure pH and assayed at zero and 144 h. In addition to the infusors, four ampoules of Lucassin were also prepared on the first day.

Two of the ampoules were used for pH measurement and assay at time zero and two were kept in the refrigerator (2–8 °C) for pH measurement and assay in triplicate at 144 h. The purpose of this procedure was to determine if there was any interaction between rubber in the infusor and terlipressin. In addition, a standard solution of terlipressin was prepared at a concentration of 0.05 mg/mL using analytical grade terlipressin. A portion of this solution was assayed by HPLC at the start and the end of the analysis on each day to determine any variability of the instrument throughout the day. The mean peak area was determined and the concentration of terlipressin remaining at each time point was determined from the calibration equation and expressed as mean ± standard deviation as a percentage of this initial concentration at time zero of the corresponding infusors and ampoules. In addition, any physical changes, such as colour of the solutions, haziness or precipitation, were assessed visually, each day to assess the physical stability.

## Results

### Assay methodology

**Figure 1:**
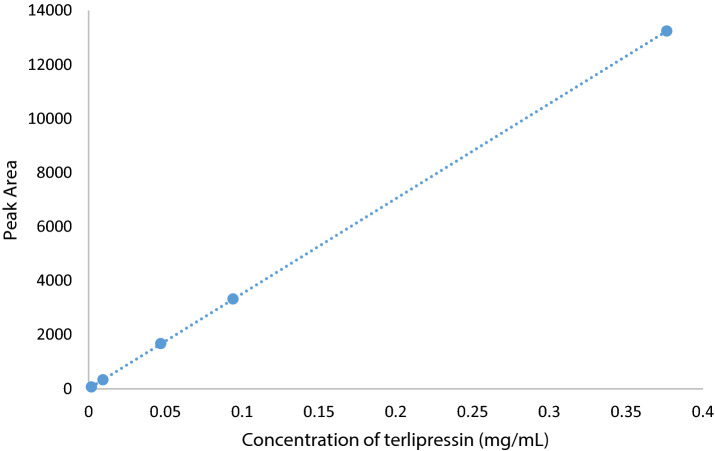
Calibration curve of terlipressin over a concentration range of 0.002–0.38 mg/mL.

The terlipressin analytical standard assayed by HPLC showed a retention time of 20.3 min. A calibration curve was created in Excel from five analytical standard concentrations (Fig. 1). The line of best fit equation was y = 35,167.9x + 7.4 (R^2^ = 1.00).

The R^2^ was 1.00 indicating a linear relationship over the concentration range 0.002 and 0.38 mg/mL. The concentration of four ampoules of reconstituted commercial terlipressin was 0.034 mg/mL and as this was within the range of the calibration curve, it confirmed the HPLC method was suitable for further study.

The intraday precision data for analytical terlipressin 0.05 mg/mL had a RSD 0.07% which was less than 2%. The interday data was collected after a second day injection and gave a value of 0.27% RSD (below the 2% limit) (Table 2).

**Table 2:** The intraday and interday precision of terlipressin standard 0.05 mg/mL.

Concentration (mg/mL)	Area		2 Days
	Day 1	Day 2	Area
0.05	1762.1	1751.1	–
0.05	1762.0	1751.1	–
0.05	1760.6	1750.2	–
0.05	1759.3	1754.7	–
0.05	1760.4	1753.0	–
0.05	1759.2	1751.2	–
Mean	1760.6	1751.9	1756.2
Standard deviation	1.26	1.66	4.76
RSD (%)	0.07	0.09	0.27

**Figure 2:**
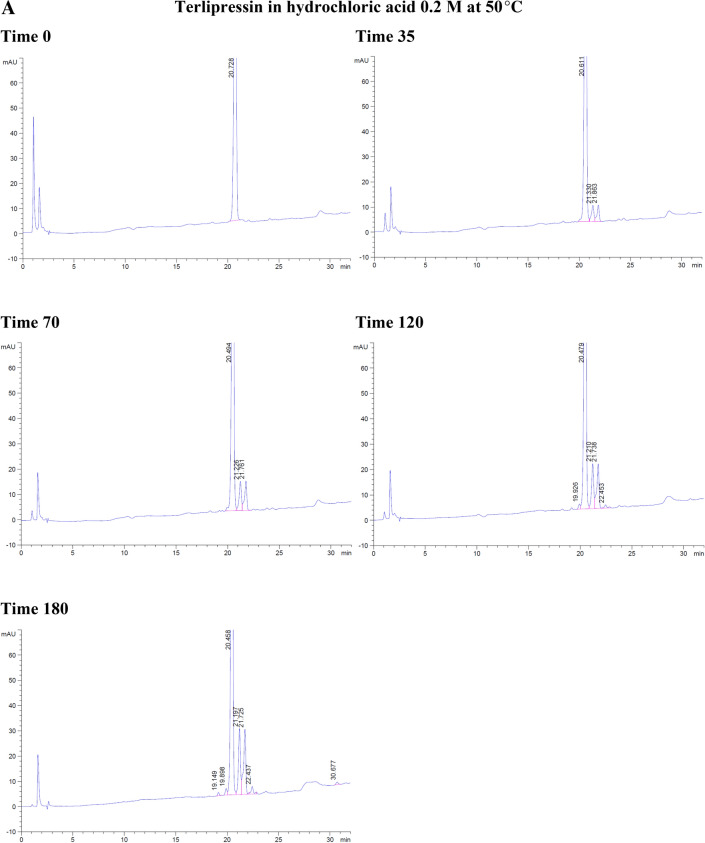

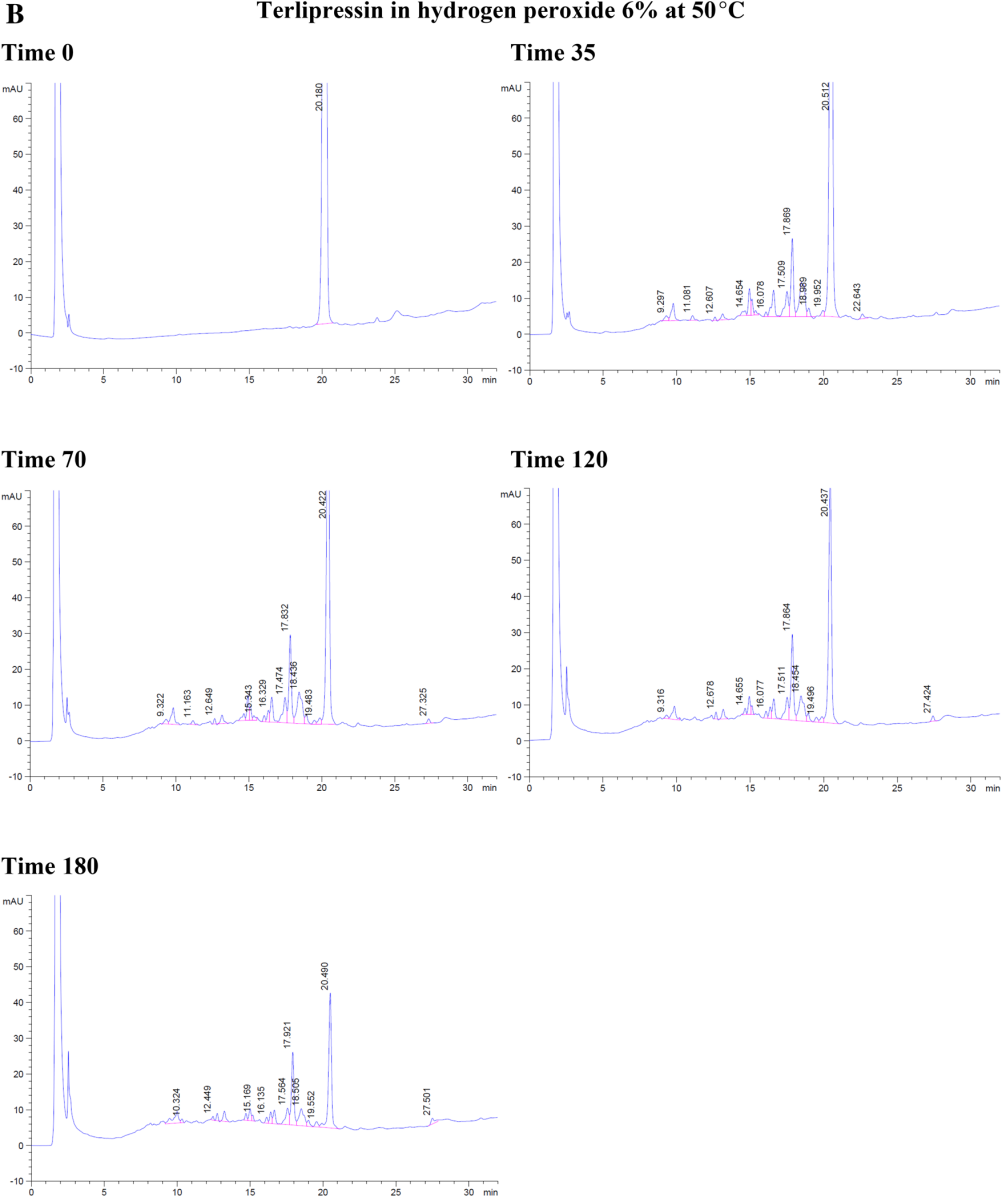

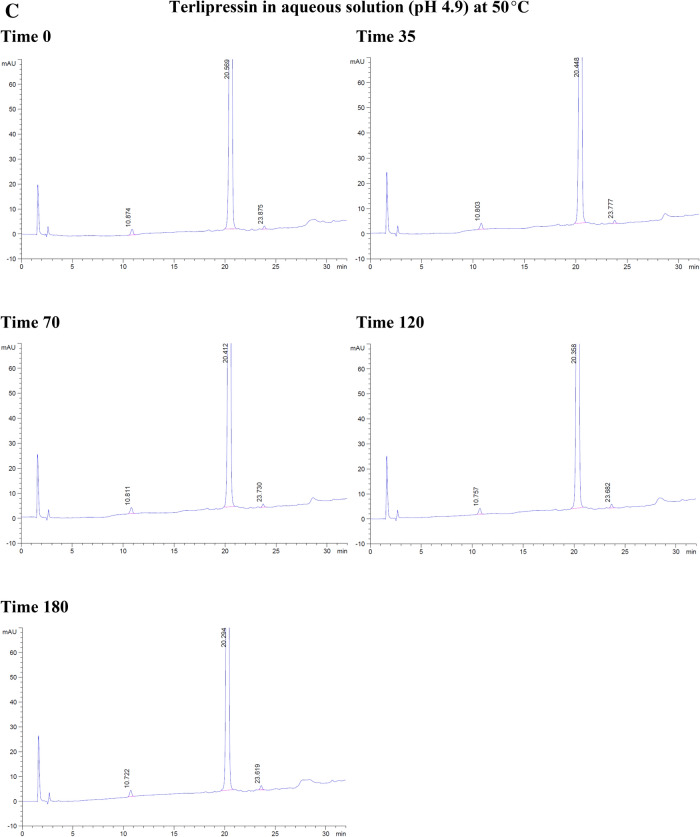

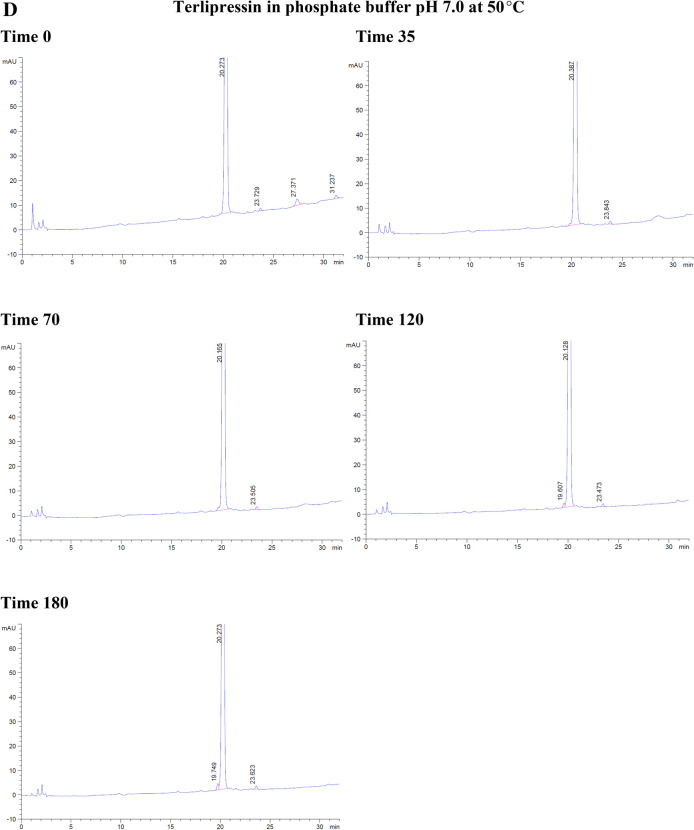
The degradation of commercial terlipressin 0.17 mg/mL in (**A**) hydrochloric acid 0.2 M from time 0–180 min, (**B**) hydrogen peroxide 6% from time 0–180 min, (**C**) aqueous solution pH 4.9 from time 0–180 min, (**D**) phosphate buffer pH 7.0 from time 0–180 min.

With the forced degradation, the commercial terlipressin samples 0.17 mg/mL showed degradation within 35 min in hydrochloric acid 0.2 M and 6% hydrogen peroxide. With hydrochloric acid, the degradation peaks were separated from terlipressin’s retention time to the right side. From 70 to 180 min, the degradation peaks increased and were separated to the left and the right of terlipressin peak (Fig. 2A).

With hydrogen peroxide, the terlipressin peak was reduced and the degradation peak was separated to the left side. At 70 min, the terlipressin peak was reduced further, which corresponded with more degradation peaks appearing on the left side. By 180 min, the terlipressin peak area decreased markedly (Fig. 2B). Both hydrochloric acid and hydrogen peroxide degradation showed no interfering degradation peaks appearing within the terlipressin retention time.

At a temperature of 50 °C, terlipressin in aqueous solution (pH 4.9) or in phosphate buffer (pH 7.0) showed no degradation peak from time 0–180 min and no interfering peak near the retention time point for terlipressin (Fig. 2C, D).

Forced degradation in hydrochloric acid decreased terlipressin concentration to 86.23% after 3 h and in hydrogen peroxide it decreased to 8.52% after 3 h (Table 3). Forced degradation in 0.2 M NaOH produced many peaks interfering with that of terlipressin, rendering the assay unsuitable at high alkaline pH values. The aqueous solution and pH 7.0 phosphate buffer assay percentages were slightly increased due to assay variation, showing no detectable degradation (Table 3).

**Table 3:** The percentage of terlipressin at each time point and each set of conditions for forced degradation at 50 °C.

Time (min)	Hydrochloric acid	Hydrogen peroxide	Aqueous solution pH 4.9	Phosphate buffer pH 7.0
0	100.00	100.00	100.00	100.00
35	95.36	41.15	100.60	101.30
70	94.65	24.73	101.80	101.61
120	88.41	15.07	100.68	100.22
180	86.23	8.52	104.11	101.14

Overall, terlipressin was degraded in hydrochloric acid and hydrogen peroxide and was stable in aqueous solution (pH 4.9) and phosphate buffer (pH 7.0) over 3 h.

### Assay of commercial terlipressin

The concentration of terlipressin remaining in the ampoules and infusors, which were refrigerated, are shown in Table 4. The stability of 0.034 mg/mL terlipressin, investigated in three separate infusors during storage in a refrigerator for 7 days, showed that the concentration of terlipressin was maintained above 90% of its initial concentration. Visual examination of the solutions in the infusors stored in the refrigerator for 7 days showed no physical changes in colour and no precipitation or haziness visually identified. Therefore, terlipressin was considered chemically and physically stable for 7 days under refrigeration.

**Table 4:** The concentration of terlipressin stored in the infusors (2–8 °C) and ampoules at different time points, t = hours.

Infusor/ampoule	Mean terlipressin concentration remaining % ± SD%				
	t = 0	t = 24	t = 48	t = 72	t = 144
1	100.00 ± 0.16	100.22 ± 0.33	99.99 ± 0.08	100.34 ± 0.03	100.61 ± 0.04
2	100.00 ± 0.08	99.70 ± 0.02	100.02 ± 0.01	100.23 ± 0.07	100.56 ± 0.10
3	100.00 ± 0.12	99.66 ± 0.14	99.87 ± 0.17	100.17 ± 0.01	100.56 ± 0.08
4	100.00 ± 0.07	–	–	–	100.59 ± 0.01
Ampoule	100.00 ± 0.04	–	–	–	98.41 ± 0.07

*SD* standard deviation.

The concentration of terlipressin remaining in each solution after an additional 24 h storage in the stability chamber at 22.5 °C at different time points is shown in Table 5. All of the solutions had terlipressin concentrations above 90% of the original concentration. Therefore, they were considered stable after a 24-h subsequent storage in the stability chamber.

**Table 5:** The concentration of terlipressin remaining in each solution after 24 h storage in the stability chamber at different time points, t = hours.

Infusor	Mean terlipressin concentration remaining % ± SD%				
	t = 0	t = 24	t = 48	t = 72	t = 168
1	100.00 ± 0.16	99.35 ± 0.08	98.29 ± 0.06	99.03 ± 0.05	100.13 ± 0.02
2	100.00 ± 0.08	98.45 ± 0.01	98.92 ± 0.02	98.71 ± 0.14	99.69 ± 0.37
3	100.00 ± 0.12	98.60 ± 0.17	99.21 ± 0.06	98.88 ± 0.09	99.66 ± 0.06

*SD* standard deviation.

The pH values of each of the four infusors ranged from 6.50–7.60 at time zero to 6.42–7.00 at 144 h. The pH of the ampoule at time zero was 4.85 and at 144 h, it was 4.88. The pH values of the solutions stored in the stability chamber for 24 h at 22.5 °C ranged from 6.75–7.19 at 24 h to 6.37–7.01 at 168 h.

## Discussion

This study investigated the stability of terlipressin stored in elastomeric infusion devices for up to 1 week at refrigerated conditions and subsequently for 24 h at 22.5 °C. Minimal loss of terlipressin concentration in infusors when stored under refrigerated conditions was evident. No colour change, haziness or precipitation in the solutions was visually evident during the study period. These findings indicate that terlipressin demonstrated physical and chemical stability under the conditions studied and analytical methods employed when stored in the infusors at 2–8 °C for 7 days and 24 h when maintained at 22.5 °C for a subsequent 24 h, mimicking patient administration conditions. Solutions that retained 90% or greater of their initial concentration were considered chemically stable.

Currently, terlipressin is administered as a slow IV bolus injection in the hospital setting^14^ although there is anecdotal evidence of increased interest in using terlipressin as a 24 h infusion. There are existing reports indicating its use as a 24 h infusion^15,18^. Cavallin et al.^15^ reported that terlipressin given as a continuous intravenous solution showed a better safety profile than an intravenous bolus injection. In that study, patients received a 1 mg terlipressin bolus followed by a 4 mg infusion over 24 h^15^. A significant reduction in portal pressure was reported compared to those treated with a 2 mg bolus and continued with 1 mg bolus injections 4 hourly over 24 h^15^. Patients injected with 2 mg/day were compared with those patients with 0.5 mg terlipressin bolus 4 hourly initially^15^. Both injections were increased gradually to 12 mg/day^15^. The results indicated that the infusion group experienced less adverse effects (35%) than the bolus group (62%)^15^. It was suggested that terlipressin given as a continuous solution was the most suitable approach for patients with HRS type 1^15^. In another study, Gerbes et al.^19^ showed that patients administered continuous terlipressin infusion achieved better results than intravenous bolus (42% vs. 35%) with low doses to minimize adverse effects. Mukhtar et al.^20^ reported that the terlipressin continuous infusion was used for 48 h in patients undergoing liver transplant. The researchers reported that these patients showed renal protection within 2 days after surgery^21^.

Terlipressin is a synthetic polypeptide drug^14^. The stability of peptides depends on their amino acid composition and sequence^21^. Peptides undergo degradation by chemical mechanisms, such as hydrolysis, deamidation and oxidation, and physical mechanisms, such as denaturation, self-association and protein aggregation^21^. Based on the percentage of terlipressin remaining following the forced degradation studies, HPLC analysis detected degradation at low pH values and in hydrogen peroxide. The temperature used for forced degradation was 50 °C which is the highest temperature suitable to allow for stress testing of polypeptide drugs owing to possible reactions occurring at high temperatures which may not occur at normal storage temperatures^22^. Rapid degradation was evident at high pH values with multiple interfering peaks rendering the assay unsuitable at high pH values. Therefore forced degradation was conducted at pH values relevant to the formulation pH range. The physical assessment of the sample solution showed no haziness or precipitation indicating protein aggregation was not visually evident during the study period.

On the other hand, the product information from the manufacturer stated that each reconstituted ampoule should have a pH range of 4.3–7.5 and contains glacial acetic acid and/or sodium hydroxide to adjust the pH^14^. In this study, solutions were prepared with 0.9% normal saline, which was not buffered. Therefore, there could be changes in the final pH of the solutions. The pH values overall showed only small changes over the course of the study but some initial pH values showed higher variation.

In a study by Kumar et al.^23^, terlipressin was assayed by HPLC using a mobile phase consisting of acetonitrile and phosphate buffer in the ratio of 35:65 v/v at a flow rate of 1.5 mL/min. The detection wavelength used in that method was 280 nm^23^. The method described in that study was not able to be repeated for this study.

### Limitations

This investigation was performed over a limited period of time. Further investigations on potential protein and peptide aggregation were not able to be performed. All the procedures were done under normal laboratory conditions. The pH of the sample solutions was not adjusted. This study investigated the stability of terlipressin at one concentration (0.034 mg/mL) in refrigerated infusors for a 7-day period. Stability at other concentrations, under other environmental conditions and for periods longer than this were beyond the scope of this study.

## Conclusion

Terlipressin reconstituted in normal saline and stored in infusors was stable for 1 week at refrigerated temperatures. Subsequent storage at 22.5 °C for 24 h also retained stability. No colour changes, haziness or precipitation were visually evident in any of the experimental samples. Therefore, terlipressin showed chemical and physical stability when stored in elastomeric infusors at 2–8 °C for 7 days and subsequently in a stability chamber at 22.5 °C for 24 h during a 7-day period. These findings suggest that terlipressin could be used in infusors as an infusion for ‘hospital in the home’ intravenous administration for up to a 7-day period.

## References

[1] Fagundes C, Ginès P (2012). Hepatorenal syndrome: a severe, but treatable, cause of kidney failure in cirrhosis. Am. J. Kidney Dis..

[2] Allegretti AS (2017). Terlipressin versus placebo or no intervention for people with cirrhosis and hepatorenal syndrome. Cochrane Database Syst. Rev..

[3] Zhang J (2019). Terlipressin for the treatment of hepatorenal syndrome: an overview of current evidence. Curr. Med. Res. Opin..

[4] Colle I, Laterre PF (2018). Hepatorenal syndrome: the clinical impact of vasoactive therapy. Expert Rev. Gastroenterol..

[5] Wang H, Liu A, Bo W, Feng X, Hu Y (2018). Terlipressin in the treatment of hepatorenal syndrome: a systematic review and meta-analysis. Medicine..

[6] Cavallin M (2015). Terlipressin plus albumin versus midodrine and octreotide plus albumin in the treatment of hepatorenal syndrome: a randomized trial. Hepatology.

[7] Neri S (2008). Terlipressin and albumin in patients with cirrhosis and type I hepatorenal syndrome. Dig. Dis. Sci..

[8] Sanyal AJ (2008). A randomized, prospective, double-blind, placebo-controlled trial of terlipressin for type 1 hepatorenal syndrome. Gastroenterology.

[9] Silawat FN (2011). Efficacy of terlipressin and albumin in the treatment of hepatorenal syndrome. World Appl. Sci. J..

[10] Boyer TD (2016). Terlipressin plus albumin is more effective than albumin alone in improving renal function in patients with cirrhosis and hepatorenal syndrome type 1. Gastroenterology.

[11] Ferring Pharmaceuticals. Terlipressin (Glypressin). MIMS Online. 2020. Available from: https://www-mimsonline-com-au.dbgw.lis.curtin.edu.au/Search/FullPI.aspx?ModuleName=Product%20Info&searchKeyword=Glypressin&PreviousPage=~/Search/QuickSearch.aspx&SearchType=&ID=94940001_2. Accessed 11 Nov 2020.

[12] Sharman A, Low J (2008). Vasopressin and its role in critical care. CEACCP.

[13] Papaluca T, Gow P (2018). Terlipressin: current and emerging indications in chronic liver disease. J. Gastroenterol. Hepatol..

[14] Mallinckrodt Pharmaceuticals. Terlipressin (Lucassin). MIMS Online. 2020. Available from: https://www-mimsonline-com-au.dbgw.lis.curtin.edu.au/Search/FullPI.aspx?ModuleName=Product%20Info&searchKeyword=lucassin&PreviousPage=~/Search/QuickSearch.aspx&SearchType=&ID=97550001_2. Accessed 11 Nov 2020.

[15] Cavallin M (2016). Terlipressin given by continuous intravenous infusion versus intravenous boluses in the treatment of hepatorenal syndrome: a randomized controlled study. Hepatology.

[16] Foy F (2019). An investigation of the stability of meropenem in elastomeric infusion devices. Drug Des. Dev. Ther..

[17] Medicines and Healthcare products Regulatory Agency (MHRA). British Pharmacopoeia. Monographs: medicinal and pharmaceutical substances/erlipressin. In: London: The Stationary Office. Available from: https://www-pharmacopoeia-com.dbgw.lis.curtin.edu.au/bp-2019/monographs/terlipressin.html?date=2019-07-01&text=terlipressin. Accessed 19 Jul 2019 (2019).

[18] Robertson M (2014). Continuous outpatient terlipressin infusion for hepatorenal syndrome as a bridge to successful liver transplantation. Hepatology.

[19] Gerbes AL, Huber E, Gülberg V (2009). Terlipressin for hepatorenal syndrome: continuous infusion as an alternative to iv bolus administration. Gastroenterology.

[20] Mukhtar A (2011). The use of terlipressin during living donor liver transplantation: effects on systemic and splanchnic hemodynamics and renal function. Crit. Care Med..

[21] Manning MC, Chou DK, Murphy BM, Payne RW, Katayama DS (2010). Stability of protein pharmaceuticals: an update. Pharm. Res..

[22] Blessy M, Patel RD, Prajapati PN, Agrawal Y (2014). Development of forced degradation and stability indicating studies of drugs—a review. J. Pharm. Sci. Anal..

[23] Kumar PM, Sreeramulu J (2010). Stability indicating RP-HPLC method for simultaneous determination of terlipressin in pure and pharmaceutical formulation. J. Chem. Pharm. Res..

